# Status and clinical influencing factors of involuntary admission in chinese patients with schizophrenia

**DOI:** 10.1186/s12888-022-04480-3

**Published:** 2022-12-21

**Authors:** Hua-Jian Ma, Yu-Chen Zheng, Yang Shao, Bin Xie

**Affiliations:** Shanghai Mental Health Center, Shanghai Jiao Tong University School of Medicine, 200030 Shanghai, P. R. China

**Keywords:** Involuntary admission, Schizophrenia, Rate, Influencing factors, China

## Abstract

**Background:**

Though controversial for its various disadvantages, involuntary admission (IA) is necessary in providing mental health care for patients suffering from schizophrenia in China. This article examines the IA rate in a representative sample, and under which circumstances are these patients more likely to be admitted involuntarily.

**Methods:**

Adult patients consecutively admitted to two typical hospitals in Shanghai between 2013 and 2014 with a diagnosis of ICD-10 schizophrenia were included. 2167 patients were included in this study. Sociodemographic and clinical data, as well as personal information of psychiatrists who made risk assessment, were collected. The whole sample was divided into voluntary and involuntary admission groups. Group comparisons were performed with SPSS 17.0, using one-way ANOVA, Wilcoxon rank sum test, Chi-squares and Logistic regression.

**Results:**

Among 2167 inpatients, the majority (2003, 92.4%) were involuntarily admitted. Clinical features, including age of patients (*p* < 0.001, OR = 1.037), lacking of insight (*p* < 0.001, OR = 3.691), were statistically significant for IA. Psychiatrist’s age (*p* < 0.001, OR = 1.042) was independently associated with IA. However, risk behaviors had dramatically affected patients’ admission status, of which the strongest predictor of IA was noncompliance with treatment (*p* < 0.001, OR = 3.597). The areas under the curve of the ROC and accuracy for the regression model were 0.815 and 0.927, respectively.

**Conclusion:**

IA patients account for a major proportion of all those hospitalized with schizophrenia in China. Insights and risk behaviors contributed the most reasons for admission status of patients. This research shed light on necessity of further qualitative studies learning detailed evaluation processes of IA and high-quality interventional studies aiming to limit the performance of IA among patients with schizophrenia.

## Introduction

Schizophrenia is a chronic mental disorder with disabling and early-occurred features [1]. In the latest national survey, weighted lifetime prevalence of schizophrenia and other psychotic disorder is 0.7% in China [2]. It’s estimated that there are more than 15 million schizophrenic patients in China, which results in heavy social and economic burden [3–5]. However, due to inadequate access of community mental health services, hospitalization remains the main way in China’s majority of families caring for mental illness patients [6, 7]. What’s worse, schizophrenic patients are more likely to be admitted involuntarily in hospital compared to other mental disorders according to previous studies [8–12].

Involuntary admission (IA) is necessary but disputed in psychiatry, due to its ethical complexity and possible multiple adverse outcomes in the future [13–17]. Discussions about its use and possible reduction are called for both professionally and politically [13, 18]. However, simply limiting the use of IA may lead to adverse consequences, for example, a stricter standard of IA would delay treatment but early intervention or rational prevention would make it more beneficial for patients [19]. Combined with high incidence of IA in schizophrenia patients and desire of reducing IA rate, we try to figure out risk factors of IA in patients with schizophrenia to provide suggestions for future work.

As many studies have revealed [7, 20–23], diagnosis of schizophrenia is one of the most influential factors of IA. This may be partly explained by the association between active psychotic symptoms and possible violent behaviors in patients with schizophrenia [24–27]. Though inconsistent results coexist [19], some reports [28–30] indicated that violence might be driven by symptoms of psychosis. Besides, lack of insight [14] would destroy patients’ competence in decision-making of hospitalization, which is also common in patients with schizophrenia in the period of onset. In general, symptoms of schizophrenia may partly explain why patients are more likely to be involuntarily admitted, but other factors might also exist and need to be figured out.

In order to apply IA rationally and protect human rights of psychotic patients, China enacted Mental Health Law of the People’s Republic of China (CMHL) on May 1st, 2013 [31]. The new law states that “inpatient treatment of mental disorders must largely be voluntary” and that IA should only be used when people with “serious mental disorders” endanger their own safety or the safety of others. It indicates that risk behavior or attempt is crucial to determining a patient’s status for admission, and patients with schizophrenia must present risk behaviors or attempts to themselves or others if they must be forced into hospital. [31] Aggressive actions or attempts would undoubtedly have an impact on the incidence of IA according to the new law. Besides, the admission process would be completed by the guardians (often family members in China) or the administrative department that refers the patients (beggars or the homeless) to the hospital for evaluation. The employer, rural village committee, or urban neighborhood committee may carry out IA procedures if the guardians decline to do so. In light of this, we speculate that the referral source may also have an impact on IA.

Before implementation of the national law, several local mental health legislations were used in local areas [32]. Shanghai was the first city to promulgate local mental health legislation in China (Mental Health Regulations of Shanghai Municipality), and “need for treatment criterion” for IA had been applied for more than ten years. The change in IA legislation was clear, dramatic and typical in Shanghai. Since pattern and influencing factors of IA in patients with schizophrenia remain unclear after the new law took effect, we try to figure these out in the study carried out in Shanghai. It is believed that status of IA in patients with schizophrenia in shanghai would be typical and representative.

In this study, rate of IA among inpatients with schizophrenia after the new law took effect was investigated, and differences of sociodemographic and clinical characteristics are compared between voluntarily and involuntarily hospitalized patients with schizophrenia to identify risk factors of IA. We hope that the present study would be the cornerstone of further study aiming to reduce the use of IA in the psychiatric institutions.

## Methods

### Patients and study sites

This was an observational study and cluster sampling was adopted. Data of medical records was collected mainly from two psychiatric hospitals in Shanghai, Shanghai Mental Health Center (SMHC, including Xuhui campus and Minhang campus) and Xuhui Mental Health Center (XMHC), between May 1, 2013, and April 30, 2014. SMHC is the only tertiary specialized psychiatric hospital with 24-hour outpatient services with 2100 psychiatric inpatient beds, as well as the largest psychiatric hospital in China. Inpatient care covered not only inhabitants in the 19 municipalities from Shanghai but also patients from other provinces. XMHC is a typical and representative district (secondary) level mental hospital (one district level hospital in each municipality and 19 ones in total) in Shanghai, which receives patients mainly from community. Persons who are suspected of having risk behaviors or intentions would be involuntarily admitted to these hospitals with consent forms signed by their family members or local civil and public security departments (only when they have no family members). As regulated in the CMHL, their situations would be estimated by a qualified psychiatrist and the final diagnosis would be made through 3-level rounds system in the hospitals [33]. In general, the IA process in China is not directly supervised by a court and the IA is usually upheld until discharge [7, 34]. But patients and family members are empowered to take the case to court if they believe the required procedures have been mismanaged. During the study period, all the data of consecutively admitted patients aged 18 years and above were extracted from medical record system. International Classification of Diseases 10th revision (ICD-10) was used for psychiatrists to make diagnosis. Patients diagnosed of schizophrenia, schizotypal and delusional disorders (F20–29) were included. Principal diagnosis was used if there was more than one diagnosis. For patients admitted more than one time within the study period, each admission of this patient was included in the study and counted separately, because they might be readmitted with different reasons and in different status (voluntary or in voluntary). Cases were excluded if the medical record data were incomplete at the time of data collection.

### Instruments and measurements

Firstly, patients’ social-demographic and clinical characteristics were collected through medical records. Socio-demographic characteristics included patients’ age, gender, employment, marriage, insurance, socioeconomic status. Clinical characteristics incorporated family history, length of illness and times of hospitalization (in SMHC, XMHC or any other psychiatric hospital). Information related to the present admission was collected such as referring agent (family member or government) and admission status (voluntary/involuntary).

Secondly, clinical symptoms collected were comprised of psychotic positive symptoms (e.g. hallucination, delusion, experiences of influence), negative symptoms (e.g. affective flattening, paucity of speech, social withdrawal and lowering of social performance), history of drug and alcohol abuse, negative life events (e.g. death of a family member, or terminal illness) or other social psychological stimulation and status of insight. Insights of patients at admission were divided into two levels (i.e. with or without insight) to get a preliminary understanding of patients’ cognitive ability to their mental state. Insights of the patients were subjectively evaluated by psychiatrists according to the following questions: *whether the patient believes that his/her abnormal experiences are symptoms; whether the patient believes his/her symptoms are attributed to mental disorder; whether he/she believes that the disorder is psychiatric; whether he/she believes that psychiatric treatment might benefit him/her; whether he would be willing to accept advice from a doctor regarding his/her treatment*.

Thirdly, personal information (i.e. gender, age, education level and title) of psychiatrists who made the clinical assessment was collected in this study. In Shanghai, an attending psychiatrist or above evaluates the situation of the patients and simultaneously records their risk status in outpatient department before admission. A risk assessment checklist for admission [35] was specified used by all psychiatrists in the mental health hospitals of Shanghai, designed according to previous researches and IA criteria and issued by the local psychiatrist association since the CMHL was enacted. When the new law took effect, all staff received training on it as well as how to use a risk assessment checklist to make sure that everyone grasped the relevant regulation and evaluation criteria. This kind of checklist record would ensure psychiatrists rational deliberative decision making based on legal criterion for IA and succinctly document grounds for admission, which comprises three sections: *harmful behaviors to self or others, current attempts of harmful behavior, history of harmful behaviors* (details see in Table 1). With the help of structured checklist, risks to self or others displayed by the patients were recorded (yes/no).

**Table 1:** Social-demographic characteristics of patients with schizophrenia

	**Voluntary admission** **(*n*=164)**		**Involuntary admission** **(*n*=2003)**		**Statistics**		
	N	%	N	%	χ^2^	*df*	*P*
**Male**	80	48.8	927	46.3	0.381	1	0.537
**Unemployed**	122	74.4	1512	75.5	0.098	1	0.754
**Marital status**					11.279	2	0.004
Unmarried	113	68.9	1112	55.5			
Currently married	40	24.4	668	33.3			
Divorced/ Widowed	11	6.7	223	11.1			
**Family history**	52	31.7	615	30.7	0.072	1	0.789
**Covered by insurance**	65	39.6	1073	53.6	11.805	1	0.001
**Low socioeconomic status**	30	18.3	416	20.8	0.569	1	0.451
**Family member referral **	163	99.4	1946	97.2	2.909	1	0.088
**Education of psychiatrists**					0.326	1	0.568
Bachelor degree and below	131	79.9	1636	81.7			
Master degree and above	33	20.1	367	18.3			
**Gender of psychiatrists: Male**	89	54.3	1101	55.0	0.030	1	0.863
**Title of psychiatrists**					3.857	2	0.145
Chief physician	60	36.6	589	29.4			
Associate chief physician	39	23.8	557	27.8			
Attending Doctor	65	39.6	857	42.8			
	Mean	SD	Mean	SD	Z		*P*
**Age of patients (years)**	32.80	11.419	38.85	15.249	-4.675		＜0.001
**Age of psychiatrists (years)**	44.94	9.409	47.35	10.513	-2.019		0.043
**Length of illness (months)**	100.24	97.483	144.63	149.130	-2.203		0.028
**Times of hospitalization**	2.24	2.835	2.70	3.508	-0.956		0.339

### Statistical analysis

The data was analyzed using SPSS version 17.0 for Windows (SPSS Inc., Chicago, IL, USA). The sample was subdivided into two groups according to their legal status at time of admission (voluntary vs. involuntary). Descriptive statistics were used to calculate rates, means, and standard deviations. Comparison of basic social demographic and clinical characteristics between two groups were performed with one-way ANOVA analysis, Mann-Whitney U test, and Pearson’s Chi-square test, as appropriate. Binary logistic regressions (forward selection) were used to determine the independent contributors to the possible risk of IA in the whole sample. The possible risk factors of IA, which were statistically significant in univariate analysis, were entered as the dependent variables, i.e. age of patients, age of psychiatrists, marital status, insurance, insight, and length of illness, risk behaviors (violent behaviors against others at admission, suicidal or self-injurious behaviors at admission, running away from home, damage in life ability, violent intentions against others, noncompliance with treatment, violent behaviors against others in the past, violent intentions against others in the past). Odds ratios (OR) and 95% confidence intervals (95% CI) were calculated. Then a receiver operating characteristic (ROC) curve was plotted to determine the predictive value of logistic regression model. The level of significance was set at 0.05 (two tailed).

## Results

There was a total of 4459 admissions of adult patients during the study period. Of all 4459 inpatients, 2427 (54.4%) were diagnosed with schizophrenia and 2167 (89.29%) of them were included in this study. Among the 2167 patients, 2003 (92.4%) were involuntarily admitted, and 164 (7.6%) were admitted voluntarily. Male patients in the sample accounted for 46.5% (*n *= 1007). Mean age of the sample was 38.39 years old (standard deviation [SD] 15.08). About 32.7% of the participants were married, 56.5% were single, and 10.8% were divorced or widowed. 75.4% were unemployed or retired.

### Social-demographic characteristics of the voluntary and involuntary admission groups

The social-demographic characteristics were compared between 164 voluntarily admitted patients (voluntary group) and 2003 involuntarily admitted patients (involuntary group) (Table 2). The IA patients with schizophrenia were more likely to be married, to have insurance, elder age, longer course of disorders, and their psychiatrists who made the assessment tended to be older than that of the voluntarily admitted patients.

**Table 2:** Clinical features of the voluntary and involuntary admission patients with schizophrenia

**Clinical features**	**Voluntary admission** **(*n*=164)**		**Involuntary admission** **(*n*=2003)**		**Statistics**		
	N	%	N	%	χ^2^	*df*	*P*
Psychotic positive symptoms *(e.g. hallucination, delusion, experiences of influence)*	118	72.0	1553	77.5	2.677	1	0.102
Negative symptoms* (e.g. affective flattening, paucity of speech, social withdrawal and lowering of social performance)*	107	65.2	1216	60.7	1.311	1	0.252
History of drug and alcohol abuse	3	1.8	63	3.1	0.889	1	0.346
Negative life events* (e.g. the death of a family member, or terminal illness)*, or other social psychological stimulation	28	17.1	392	19.6	0.605	1	0.437
Lack of insight	65	39.6	1495	74.6	92.109	1	＜0.001

### Clinical features and risk assessment of the voluntary and involuntary admission groups

Tables 3 and 1 presented comparison of clinical features and risk assessment between the voluntary and involuntary groups. Compared to voluntary admission patients, IA patients were more likely to be lack of insight, to have harmful behaviors to self or others, damage in life ability, violent intentions against others, not comply to treatment, violent behaviors or intentions against others in the past (more than one week ago), and to run away from home or wander aimlessly in the past (all *p* < 0.05).

**Table 3:** Risk assessment comparison of the voluntary and involuntary admission patients with schizophrenia

**Risk assessment checklist**	**Voluntary admission** **(*n*=164)**		**Involuntary admission** **(*n*=2003)**		**Statistics**		
	N	%	N	%	χ^2^	*df*	*P*
**Harmful behaviors to self or others in the immediate past**							
Violent behaviors against others at admission (or in the past week)	25	15.2	637	31.8	19.590	1	＜0.001
Suicidal or self-injurious behaviors at admission (or in the past week)	12	7.3	339	16.9	10.308	1	0.001
Running away from home, which results in injury or having difficulty in going home alone at admission (or in the past week)	24	14.6	571	28.5	14.647	1	＜0.001
**Potential risk of harmful behavior**							
Damage in life ability * (Be unable to take care of oneself partially or completely; * * Refuse to eat or be unable to arrange one’s diet properly, resulting in apparent body weight decreasing , electrolyte or metabolic disorder)*	34	20.7	781	39.0	21.540	1	＜0.001
Violent intentions against others * Verbal threats to others at admission (or in the past week) (e.g. reveal this attempt in words or prepare relevant tools) at admission (or in the past week)*	33	20.1	852	42.5	31.521	1	＜0.001
Noncompliance with treatment * (Be unable to accept treatment for one’s disease at the affection of mental disorder)*	20	12.2	823	41.1	53.243	1	＜0.001
Suicidal/self-injury intentions * (e.g. reveal this attempt in words or prepare relevant tools) at admission (or in the past week)*	22	13.4	284	14.2	0.073	1	0.787
**History of harmful behaviors**							
Violent behaviors against others in the past ^a^	18	11.0	467	23.3	13.286	1	＜0.001
Violent intentions against others in the past * (Verbal threats to others, or attempt to hurt others , e.g. reveal this attempt in words or prepare relevant tools)*	29	17.7	604	30.2	11.403	1	0.001
Suicidal or self-injury behaviors in the past	21	12.8	233	11.6	0.201	1	0.654
Running away from home in the past * (Wander aimlessly resulting in self-injury or having difficulty in going home alone in the past)*	21	12.8	401	20.0	5.032	1	0.025
Suicidal/self-injury intentions in the past * (e.g. reveal this attempt in words or prepare relevant tools)*	8	4.9	90	4.5	0.052	1	0.820

^a^In the past means more than one week ago

### Predictors of admission status in patients with schizophrenia

**Fig. 1:**
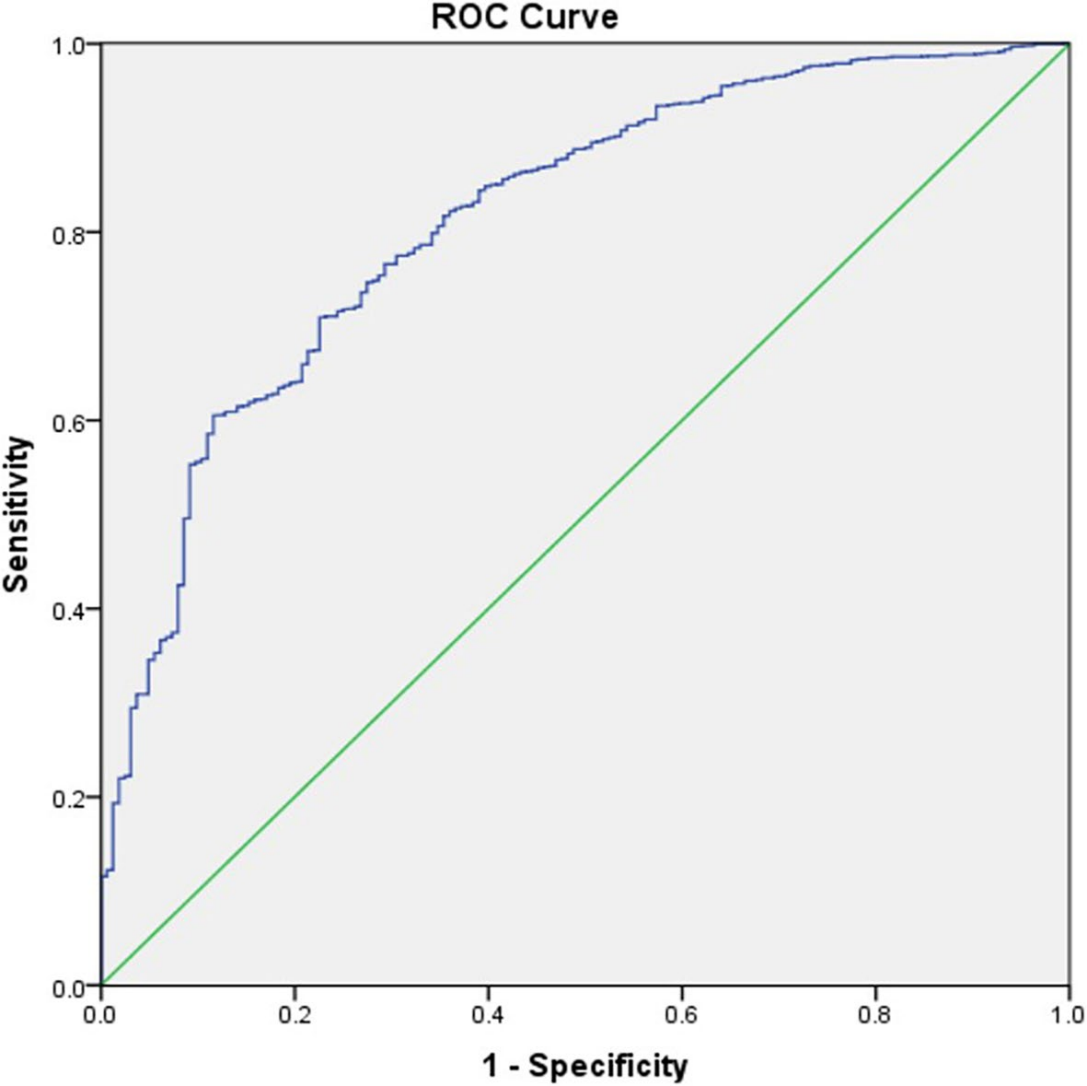
Receiver operating characteristics (ROC) curve of logistic regression prediction model for IA patients with schizophrenia

The results of forward stepwise multivariate logistic regression analysis on admission status were presented in Table 4. The independent variables entered in the model were age of patients, age of psychiatrists, marital status, insurance, risk behaviors, insight, and length of illness. Predictors of involuntary admission in this model were age of patients (*p* = 0.001; odds ratio = 1.037), age of psychiatrists (*p* = 0.001; odds ratio = 1.042), suicidal or self-injurious behaviors (*p* = 0.007; odds ratio = 2.363), running away from home/wander aimlessly (*p* = 0.046; odds ratio = 1.611), damage in life ability (*p* = 0.006; odds ratio = 1.812), violent intentions against others (*p* = 0.001; odds ratio = 2.790), not comply to treatment (*p* = 0.001; odds ratio = 3.597), lack of insight (*p* = 0.001; odds ratio = 3.691). The ROC curve in Fig. 1 showed a good performance where the AUC of the ROC was 0.815 (95% CI, 0.781–0.848), and the accuracy of this model was 0.927.

**Table 4:** Independent contributors to IA status (multiple logistic regression analysis)

	**B**	**S.E.**	**Wald**	**Sig.**	**Exp(B)**	**95%C.I.**
Age of patients (years)	0.036	0.007	27.420	＜0.001	1.037	1.023-1.051
Age of psychiatrists (years)	0.041	0.010	18.503	＜0.001	1.042	1.023-1.061
Suicidal or self-injurious behaviors	0.860	0.320	7.204	0.007	2.363	1.261-4.427
Running away from home / Wander aimlessly	0.477	0.239	3.982	0.046	1.611	1.009-2.573
Damage in life ability	0.594	0.217	7.485	0.006	1.812	1.184-2.774
Violent intentions against others	1.026	0.214	22.909	＜0.001	2.790	1.883-4.247
Noncompliance with treatment	1.280	0.257	24.791	＜0.001	3.597	2.173-5.954
Lack of insights	1.306	0.176	54.800	＜0.001	3.691	2.612-5.215

## Discussion

As far as we know, there is few studies about the prevalence and risk factors of IA in patients with schizophrenia following the implementation of CMHL. It is revealed in our study that inpatients with schizophrenia accounted for about half of all the patients (54.4%), which is consistent with previous studies at home and abroad [7, 25, 36]. Because rate of inpatients with schizophrenia is larger than those with other diagnoses and the point prevalence of schizophrenia rose with the urbanization in China [6], it is particularly important to take precautionary measures for these people avoiding hospitalization.

Meanwhile, the high proportion of unemployment (75.4%) and single status (67.3%) in this study further hints that social maladjustment was a problem for the majority of the patients with schizophrenia. This phenomenon is not rarely seen in China and other countries [4, 37], which could be partly explained by schizophrenia-related symptoms as well as social discrimination and a lack of social support [36, 38]. Promoting schizophrenia patients’ integration into society and providing them with extensive assistance through a variety of interventions is crucial to reducing the likelihood of IA recurrence. For instance, our government may encourage the hiring of patients in remission in the public and private sectors by setting hiring quotas or offering incentives to businesses, as well as expanding opportunities for employment-related training.[39].

Majority of schizophrenic inpatients (92.4%) were involuntarily admitted in this study, which is consistent with our survey [10] of 2,333 patients in 2003 (93.2%). However, the proportion was larger than that of many previous studies (29.5-64%) [7, 20, 21, 40, 41]. As stipulated in our former report [9], IA rate was generally decreasing after the CMHL was implemented, while IA rate of schizophrenia remained stable compared to that of other diagnosis. Recent systematic reviews [42] also found that IA rate in China remained high, particularly for schizophrenia, much higher than overall IA rate in western countries (43.0%) [43]. In some country, IA rate in patient with schizophrenia is also relatively high, i.e. 80.7% in one study in Norway [25]. The issue might be considered in the context of very country. The contribution of reducing stigma across Shanghai cannot be overlooked. By educating the public and passing new local legislation on mental health, the government and health authorities have made enormous efforts to reduce the stigma associated with serious mental illness. Patients and their families are more likely to seek mental health care now that the legal landscape and social climate surrounding serious mental illness are changing.[42] Shanghai’s community has grown somewhat, but given the city’s vast population, it is still insufficient. Additionally, since the majority of the city’s psychiatric beds are located in hospitals like the SMHC, the amount of resources allocated for mental health care needs to be raised. [44] To clarify the precise causes of the high IA rate in patients with schizophrenia, this still require further exploration.

Assessing patient’s demographic and clinical characteristics can help recognize patients who are at risk of IA. Studies from different countries deem that predictors of IA would vary between countries, which might be explained by differences in cultural factors [45], factors related to social services [46], legal frameworks or procedures [13, 21]. Therefore, IA influencing factors in patients with schizophrenia found in this study require concrete analysis according to concrete circumstance to make it clear what need be taken into consideration in the future policy. We set up a regression model to predict the risk of IA in schizophrenic patients. ROC curves demonstrated these factors listed in Table 4 had comparable accuracy at predicting IA (AUC were 0.815).

In the aspect of clinical characteristics, lack of insight (*p* = 0.001; odds ratio = 3.691) was the most influential factor on IA status in patients with schizophrenia. Lack of insight was also found to be a significant factor in IA in many studies, and it was thought that poor insight would postpone therapy and increase the likelihood of harmful behavior toward oneself or others. [7, 47, 48] It also cannot be neglected that psychiatrists’ risk assessment in Shanghai might be influenced by the former IA criteria [49] of the Shanghai Municipality Mental Health Regulations. The very local mental health regulations had been implemented for 10 years, which stipulated that severe lack of insight is one of the criteria for IA. The previous regulation might influence psychiatrists’ assessment and evaluation on IA in practice to some extent.

Of all the factors in the risk assessment checklist, “noncompliance with treatment” (*p* = 0.001; odds ratio = 3.597), “violent intentions against others” (*p* = 0.001; odds ratio = 2.790) and “suicidal or self-injurious behaviors” (*p* = 0.007; odds ratio = 2.363) were the top three most influencing factors on IA. Need for and acceptance of treatment is one of the four elements of insight, which might partly explain poor treatment compliance to be the other most important factor of IA in patients with schizophrenia. Poor treatment compliance mainly refers to low medication adherence in patients with mental illness. Early findings [40, 50] support that lower medication compliance would lead to a greater need for IA and re-hospitalization. A meta-analysis in 2016 [51] shows the prevalence of aggressive behavior (verbal aggression, physical aggression toward others or himself/herself, and aggressive behavior toward objects) among schizophrenia patients ranges from 15.3 to 53.2%. Some reports suggest that percentage of violent behaviors or intentions in patients with schizophrenia is higher than that in other diagnosis [36], and agitation would occur throughout the entire course of the disease. The high proportion of IA might be related to the aggressive behavior in patients with schizophrenia. As many studies revealed [20, 52, 53], perceived violence (risk of dangerousness to self or others) is a central factor in psychiatrists’ judgments regarding IA, and this is true especially under the risk criteria provisions on IA in China. Psychiatrists’ assessment would reflect their evaluation of patients as well as their attitude and understanding of risk criteria on IA [54, 55]. In other words, the three factors above might be the most important for clinicians on IA decision making.

As for demographic factors, mean age of IA patients with schizophrenia were elder than that of voluntarily admitted ones, which was an independent influencing factor for IA status (*p* = 0.001; odds ratio = 1.037). This is consistent with many reports in both Asian and Western studies, which demonstrated higher rates of IA among the elders [26, 40]. However, some research [56] presents the opposite results. The finding in our study might be explained by the fact that older patients with schizophrenia generally have poorer insights [47, 57] and gradual destruction of social functions following its progressive course, and severity of the disorder grows with age, which would lead to higher possibility of involuntary hospitalization. We cannot exclude the assumption of psychiatrists’ habitual practice on IA decision-making [8, 49] when facing patients with longer course of schizophrenic patients. It’s also interesting to note that psychiatrists who made risk assessment for IA patients with schizophrenia were also elder than those for voluntary admitted patients (*p* = 0.001; odds ratio = 1.042). This phenomenon might be regarded to the following factors. On the one hand, older psychiatrists usually have higher professional qualifications and treat patients with more severe symptoms who would be more likely to be involuntarily admitted. On the other hand, our previous study [49] found that clinicians might show more arbitrarily attitudes in the admission process and prone to use their discretions in making clinical decision due to conservative values of the older generations [52]. Psychiatrists’ characteristics and assessments had significant impacts on IA of schizophrenia patients [58], which suggested the importance of systematic training to psychiatrists and third-party review systems. Establishment of regular and effective monitoring processes focusing on involuntary psychiatric hospitalizations would be an important step forward.

This study tries to find independent influencing factors between voluntary and involuntary admission in patients with schizophrenia, and attempts to make suggestions for future policy implementation in Shanghai and even China according to these factors. There are several limitations of this study should be noted. Firstly, the sample of this study is from only one city in China, but Shanghai is a typical city and representative for its change of legislation from “treatment criteria” to “risk criteria”. The present result still should be considered as preliminary and need to be confirmed with further studies. Secondly, this is quantitative research, and more details of risk assessment, such as evaluation process and attitudes and other consideration of decision-making, should be verified in future qualitative studies. Besides, the statistics were less precise because we only performed a retrospective analysis and did not discuss the connection between the severity of the symptoms and IA due to our inability to get more precise data. Due to the study method, other important variables were also missing, such as traumatic life events or other types of social psychological stimulation. Thus, longitudinal and prospective studies are required to support the current finding.

## Conclusion

The present study revealed a high rate of IA in patients with schizophrenia. The principal contribution of this study was identification of independent influencing factors of IA in patients with schizophrenia, including older age of patients and psychiatrists who made assessment, lack of insight, noncompliance with treatment, risk behaviors, such as violent intentions against others, suicidal or self-injurious behaviors, running away from home/wander aimlessly and damage in life ability. To reduce IA rate of schizophrenia patients, more attentions should be paid to those risk factors and preventive measures should be made once these factors occurred. Besides, regular training should be made to make psychiatrists risk assessment more reasonable and effective. Further qualitative studies are needed to learn more about evaluation process of IA and high-quality interventional studies are needed to make it possible the reduction of IA rate in patients with schizophrenia.

## Data Availability

The datasets generated and analysed during the current study are not publicly available due confidentiality reasons but are available from the corresponding author on reasonable request.

## Abbreviations

IA: Involuntary admission
CMHL: Mental Health Law of the People’s Republic of China
SMHC: Shanghai Mental Health Center
XMHC: Xuhui Mental Health Center
